# An Automated System for Classification of Chronic Obstructive Pulmonary Disease and Pneumonia Patients Using Lung Sound Analysis

**DOI:** 10.3390/s20226512

**Published:** 2020-11-14

**Authors:** Syed Zohaib Hassan Naqvi, Mohammad Ahmad Choudhry

**Affiliations:** 1 Department of Electronics Engineering, University of Engineering and Technology, Taxila 47080, Pakistan; 2 Department of Electrical Engineering, University of Engineering and Technology, Taxila 47080, Pakistan; dr.ahmad@uettaxila.edu.pk

**Keywords:** chronic obstructive pulmonary disease, lung sounds, pneumonia, quadratic discriminant analysis, feature extraction, empirical mode decomposition, discrete wavelet transform

## Abstract

Chronic obstructive pulmonary disease (COPD) and pneumonia are two of the few fatal lung diseases which share common adventitious lung sounds. Diagnosing the disease from lung sound analysis to design a noninvasive technique for telemedicine is a challenging task. A novel framework is presented to perform a diagnosis of COPD and Pneumonia via application of the signal processing and machine learning approach. This model will help the pulmonologist to accurately detect disease A and B. COPD, normal and pneumonia lung sound (LS) data from the ICBHI respiratory database is used in this research. The performance analysis is evidence of the improved performance of the quadratic discriminate classifier with an accuracy of 99.70% on selected fused features after experimentation. The fusion of time domain, cepstral, and spectral features are employed. Feature selection for fusion is performed through the back-elimination method whereas empirical mode decomposition (EMD) and discrete wavelet transform (DWT)-based techniques are used to denoise and segment the pulmonic signal. Class imbalance is catered with the implementation of the adaptive synthetic (ADASYN) sampling technique.

## 1. Introduction

**Figure 1:**
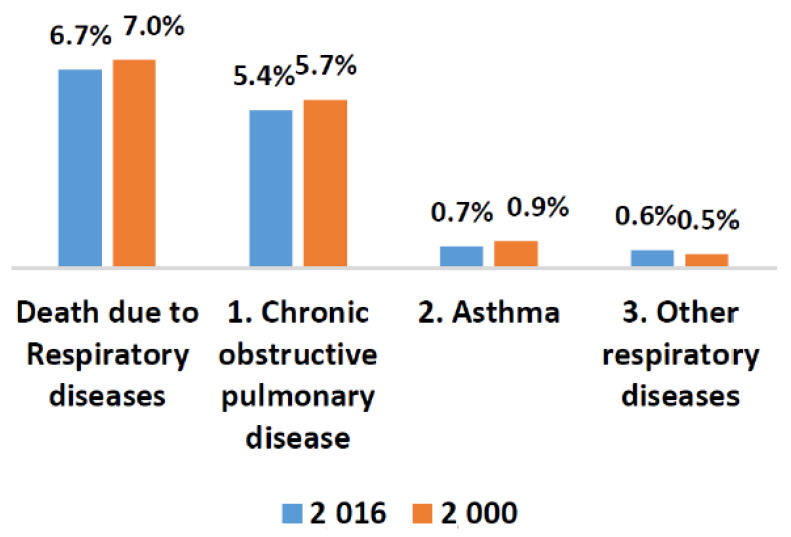
WHO statistics about worldwide deaths due to respiratory issues in 2000 and 2016.

**Figure 2:**
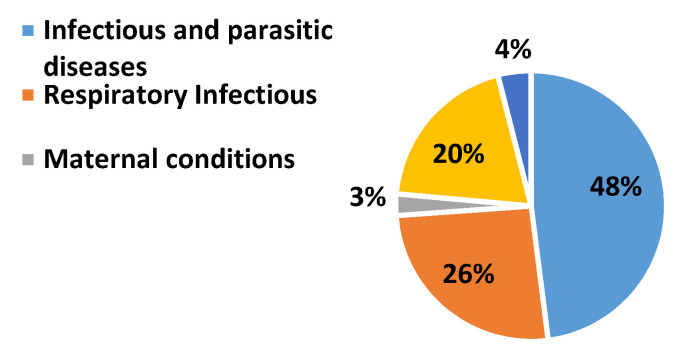
WHO statistics about worldwide death due to different respiratory issues in 2016 [3].

Pulmonary abnormalities encompass various lethal diseases. Chronic obstructive pulmonary disease (COPD) and pneumonia are treatable pulmonic illness with early diagnosis and proper prevention. Pneumonia is a pulmonary abnormality which can be caused by virus, bacteria, or fungi. Infections. COPD subjects are also vulnerable to a high risk of pneumonia. The subjects who develop pneumonia are more likely to die. According to United Nations Children’s Fund (UNICEF), half of the morbidity from the recorded 5.9 million under-five deaths has caused due to infectious illnesses and conditions in which pneumonia lies at premier rank in 2015 [1]. It is a challenging task to distinguish pneumonia and acute exacerbation of COPD as both displays the same symptoms [2]. Exacerbations as well as cough are commonly found in both COPD and pneumonia patients. The frequent global pervasiveness of COPD is also evidenced by the WHO statistics. A reduction of 0.3% is estimated in global mortality in 2016 when it is compared with estimates of 2000 as shown in Figure 1 and Figure 2 [3]. Most importantly, COPD patients are vulnerable to a high risk of pneumonia and other related diseases like bronchitis. Due to viral, bacterial, or both infections, COPD lies at the third position in global mortality characterized by frequent exacerbations. It damages the lungs and blocks airways with mucus does not allow them to function properly. Underestimation of the death rate due to COPD often occurs because of insufficient knowledge and variations in the diagnostic standards for the disease. The false diagnostic rate may be greater than 70% [4]. Qualitative analysis is an alternative approach but it is purely based on the expertise of medical experts. Lack of experience can cause irreversible loss to a patient. In the current era, the field of computer technology has made tremendous advancements in the early and rapid diagnosis of various adventitious sounds and pulmonary diseases from lung sound (LS) data banks [5]. Imaging pathologies like magnetic resonance imaging (MRI) and computerized tomography (CT) has provided the best diagnosis for pulmonary issues. Conversely, the cost of its machines, exposure to harmful radiations, and inconvenient to deploy in rural and far-flung areas are few bottlenecks.

Signal analysis in conjunction with the machine learning (ML) approaches on LS analysis can provide better results for COPD and pneumonia diagnosis [6,7]. Feature engineering to existing ML prototypes can be a useful way to differentiate between the same adventitious sounds of different pulmonic diseases. These bottlenecks demand an efficient, economic, convenient, and noninvasive diagnostic methodology for the identification of pulmonic disease [8]. The need for an hour is to design a prototype or a system capable of accurately detect and classify pulmonary diseases like COPD and pneumonia from a simple and less intrusive modality.

## 2. Literature Review

Recently, machine learning (ML) schemes have been reported to identify a single lung disorder from lung sound (LS) analysis of adventitious sounds [9]. Different schemes devised to analyze the LS analysis via electronic auscultation is a better alternative approach to trace pulmonary diseases against invasive and costly imaging diagnostic techniques [10,11,12,13]. Numerous studies performed on pulmonary issues for early diagnosis but these abnormalities are quite complex and complicated. High cost to build large scale and well-labeled episodes are major constraints to realize this approach. On the contrary, limited training data will raise the model overfitting and low-reliability issues [14]. Novel predictive prototypes can incorporate both symptoms and physiological signals are being tested in telemonitoring interventions with positive outcomes.

However, its validation needs further investigation in the diagnostics of COPD and the identification of multiple lung diseases [15]. In time and accurate diagnosis can lower the death risk, but the subjective nature of adventitious sounds like coughs has led to high complexity in the detection of pneumonia, COPD, and other lung illnesses [16,17].

According to the literature review of related research work carried out in the last decade, most of the researchers have contributed to devising methods for diagnosing pulmonary issues via LS analysis, but a lot of efforts are still needed in this research area. Targeting single or multiple lung diseases, changes in focus groups having lung abnormality, to measure the intensity of a particular lung disease only, etc. are a few constraints that require attention. To the best of our knowledge, COPD and pneumonia LS is hardly addressed collectively in the context of LS analysis via digital signal processing (DSP) and ML techniques, so a literature review was performed targeting the studies focused on LS analysis for detection of pulmonary abnormalities, i.e., COPD and pneumonia particularly and other related lung illnesses in general.

A hybrid classifier is developed for a handheld device combined with the support vector machine (SVM), a random forest (RF), and a rule-based system to provide an advanced characterization scheme for COPD episodes in real-time. In this study, extensive data sets are required to refine the rule-based system. The data utilized in this research has missing values which are handled through two sub-algorithms to interpolate the missing information. However, the missing levels of the patient’s information in the present dataset is a constraint of the presented system. Moreover, the focus on adventitious LS analysis could enhance the system reliability but the cough sound is only monitored in this research [11]. Cough analysis also provided an alternative way for the diagnosis of rapid childhood pneumonia. It is implemented by the logistic regression (LR) classification method for the identification of pneumatic and non-pneumatic subjects, i.e. asthma, and bronchitis. The classification is performed on the statistical and wavelet features. The author highlighted that only cough is not enough to diagnose pneumonia efficiently. This gap could be the reason for the low specificity of the system and required investigation [12,13]. A few authors have designed a pneumonia screening system from LS. Features are extracted from wavelet transform (WT), and power spectrum density (PSD). The thresholding of skewness, kurtosis, and statistical analysis is performed to recognize the pneumonia subjects from cough analysis. The researchers are reluctant to claim the maximum authenticity of the proposed system as a large data set could change the thresholding estimates to differentiate pneumonia and normal subjects [17,18,19]. In [20], the statistical analysis of COPD and pneumonia LS is performed to design a detection system for multiple lung diseases. The research highlighted the significant differences in various features of focused classes. These features include harmonic-to-noise ratio, pitch, and amplitude perturbation. Furthermore, the implication of these features to perform disease classification required attention. In another research, the spectrum of wheezes, crackles, and stridor was estimated to authenticate its variation in focused groups affected by nine different pulmonic diseases. These diseases include pulmonary edema, asthma, viral bronchitis, acute asthma, tuberculosis, rheumatoid, pneumonia, epiglottis, and laryngomalacia. In this research work, authentication is made on limited LS data in case of each disease which is insufficient. Pulmonary issues are comprised of high complexities and equally dependent on age, gender, and other factors. The effect of diverse focus groups in each disease is overlooked which could vary the spectrum position of abnormal LS in diverse bands with the same illness [21]. Multichannel LS signal is investigated for the classification of asthma, COPD, and normal classes. Statistical feature extraction form sub-bands of the PSD and artificial neural network (ANN) classifiers provide significant outcomes on self-collected LS data. The proposed method demonstrated the low specificity and sensitivity as compared to most of the latest research work and required authentication on real-time or authentic data. [22]. The intensity of asthma is measured via wheeze analysis. The wheezing sound possessed different power spectral distributions in different bands according to severity levels. K-nearest neighbor (KNN) performed better than the ensemble (ENS) and SVM. The research work was based on a simulation environment without any realization in real-time. In special cases, wheeze may even absent in asthma patients. The data collection was made entirely from asthmatic subjects, however, the same adventitious sound is associated with COPD subjects which can affect the system accuracy in practice [23,24]. In another research article, a COPD diagnosis technique was based on the transfer learning (TL) approach referred to as a balanced probability distribution (BPD). The novel design accomplished better predictive capacity generally, even for a small COPD subject sample size and related common diseases. These common diseases included asthma, bronchitis, pneumonia, chronic bronchitis, and emphysema. This study mainly focused on applying the knowledge graph method on some COPD datasets and twenty-five electronic medical records. The maximum data required for the system limits its scope to adults only. Persons with disabilities in communication and pediatrics are unable to report dry throat, fatigue, itching, tongue coating, aster, and confidence features as reported in the research article for proper diagnosis. If any symptom or feature is reported due to human error then the proposed system is unable to diagnose properly. It emerges the need for an automatic, economic, and non-invasive system for the diagnosis of COPD [25,26,27]. Another study is made for the identification of pneumonia and asthma subjects from cough sound analysis, specifically focused on the pediatric population. The proposed study implemented the Mel frequency cepstral coefficient (MFCC), non-Gaussianity score, and Shannon entropy features to design the diagnostic system with the ANN technique. The system is entirely based on cough analysis to detect crackles sound. Clinically, it is not specific to pneumonia only. Other symptoms like a fever could enhance the specificity of the pneumonia diagnosis. Moreover, 44.4 % of the asthmatic subject also possessed the same issue which highlighted the need for a large database for reliable diagnosis [7]. Recently, a research article is published in which a convolutional neural network (CNN) is implemented on the LS database of the international conference on biomedical and health informatics (ICBHI). But the research work only focused to classify the adventitious sounds found in various pulmonic illnesses [28]. In another research, a novel approach called variational convolutional autoencoder is presented for unbalanced data and implemented on the same database [29].

Tremendous efforts have been made to identify COPD and pneumonia particularly, and some other lung illnesses in general from common adventitious sounds. Digital respiratory sounds provide valuable information for telemedicine and smart diagnostics in a non-invasive pathological detection way by an application of signal processing. Therefore, a comprehensive investigation is needed to devise a technique for the identification of COPD and pneumonia from common adventitious lung sounds. It is worth mentioning the important aspects to develop an efficient, robust, and reliable system for diagnosis of pulmonary pathologies, particularly COPD and pneumonia. It includes:

(i) Data mining to extract the relevant and significant data of LS signals which should help to develop the diagnosis methods for COPD, pneumonia, and healthy LS.
(ii) The design of the diagnosis method must be with simple statistical features that should not burden the system with computational cost and acquaint its performance with significant robustness.
(iii) Investigation of minimum significant features required can be prolific to perform the classification of COPD, pneumonia, and healthy LS.
(iv) Performance analysis of various classification methodologies would be required on selected features that are computationally smart.

## 3. Materials and Methods

**Figure 3:**
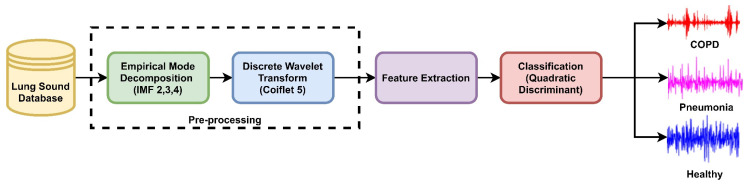
The proposed method for diagnosis of COPD, pneumonia, and healthy LS.

The time and spectral characteristics of lung sound analysis can be used to differentiate between lung sounds. There is motivation to use the cepstral-based features for the identification of adventitious LS along with time spectral and other features. As sound is a common factor in speech and LS it is expected that these features can outperform along with others like in speech signal classification. The proposed diagnostic technique is constituted of four stages: (i) data acquisition from the database, (ii) preprocessing, (iii) feature extraction, and (iv) classification. The proposed method for diagnostics of COPD, pneumonia, and healthy LS is presented in Figure 3. ROI extraction is carried out through the EMD technique to keep the domain the same and avoid information loss in LS analysis. The intrinsic mode functions encompassing the low frequencies are selected to reconstruct the LS signals of COPD, pneumonia, and healthy subjects. After denoising, features from cepstral, time, and spectral-domain are fused to investigate the performance of the proposed method on different machine learning algorithms for the classification of COPD, pneumonia, and healthy subjects.

### 3.1. Database

**Figure 4:**
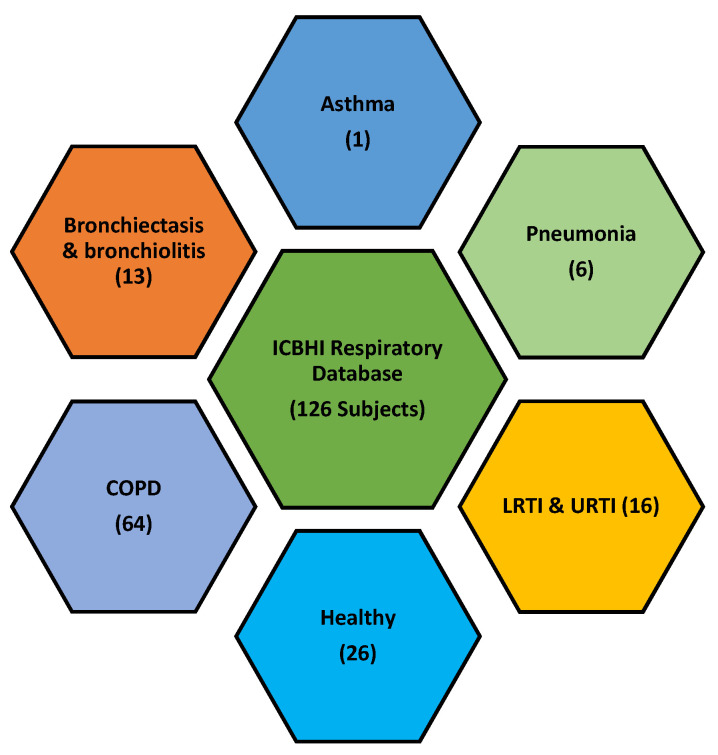
ICBHI respiratory database comprised of LS from various pulmonary pathologies.

LS data of healthy and unhealthy subjects from a public respiratory sound database of the International Conference on Biomedical and Health Information (ICBHI) is used [30]. It was compiled for an international competition, the first challenge of IMBE’s International Conference on Biomedical Health Informatics. The database comprises 920 LS recordings from 126 healthy and unhealthy subjects. The unhealthy subjects included patients with COPD, upper respiratory tract infections (URTI), bronchiectasis, asthma, and pneumonia patients, as shown in Figure 4.

In this research, 703 LS data sets of COPD, pneumonia, and a healthy subject are used. The selected data has a standard sampling frequency i.e. 44.1 kHz. Table 1 presents the information about recording equipment used to develop the LS database for research. Table 2 lists the demographic information of the focused section of the ICBHI database which consists of COPD, pneumonia, and healthy LS.

### 3.2. Pre-Processing and Segmentation

Preprocessing is a first and critical step in signal processing. It involves the removal of the baseline wander and high-frequency noise along with other artifacts that can corrupt the acquired signal. Heart sound, muscles, and skin artifacts are common interrupts. The database used in the research also lacks information about confounding noise sources. Therefore, empirical mode decomposition (EMD) [31] and discrete wavelet transform (DWT) [32] techniques are used to segment and remove the noisy portion of the signal.

**Figure 5:**
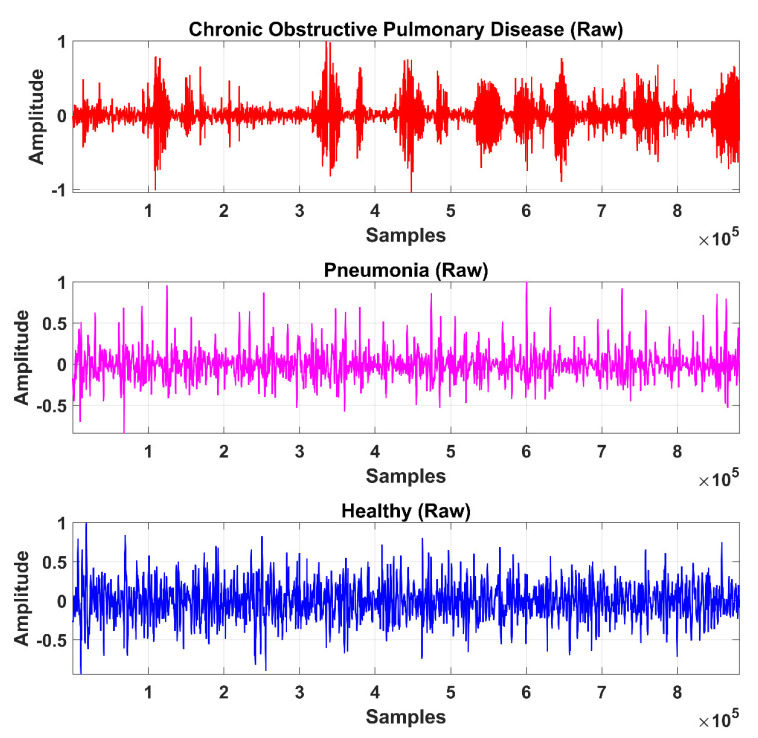
Time-domain graphical representation of an LS (raw) signal from the COPD, pneumonia, and healthy class.

**Figure 6:**
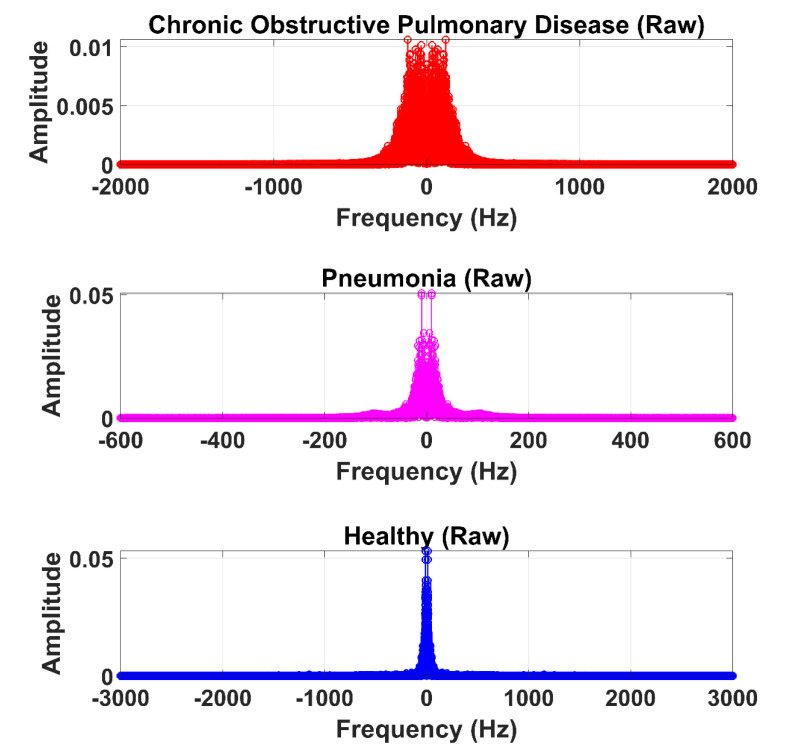
Frequency domain graphical representation of an LS (raw) signal from the COPD, pneumonia, and healthy class.

The LS signal (raw) is demonstrated in Figure 5 and Figure 6 in the time and frequency domain, respectively.

(i) Empirical Mode Decomposition

Empirical Mode Decomposition (EMD) is used in several kinds of research to decompose the signal and extract the RIO. In the EMD technique, the signal is decomposed into its components represented by an intrinsic nature function known as intrinsic mode functions (IMF). It has only one extreme between zero crossings and comprises zero mean value. An IMF is defined as a function which satisfies the following conditions:

(a) In the whole data set, the number of extreme and the number of zero-crossings must either be equal or differ at most by one.
(b) At any point, the mean value of the envelope defined by the local maxima and the envelope defined by the local minima is zero.

If L[n] is the acquired LS signal, then $m_{1}$ be the mean of its upper and lower envelopes estimated from a cubic-spline interpolation of local maxima and minima. Therefore, the first IMF can be calculated by Equation (1):

(1) $$IMF_{1}=L\left[n\right]-\;m_{1}$$

By repeating the same step according to Equation (1), IMF2 can be calculated by Equation (2):

(2) $$IMF_{11}=\;IMF_{1}-\;m_{11}$$

Generalizing the procedure, Kth IMF may be calculated from Equation (3):

(3) $$IMF_{1\left(k-1\right)}-\;m_{1k}=\;IMF_{1k}$$

**Figure 7:**
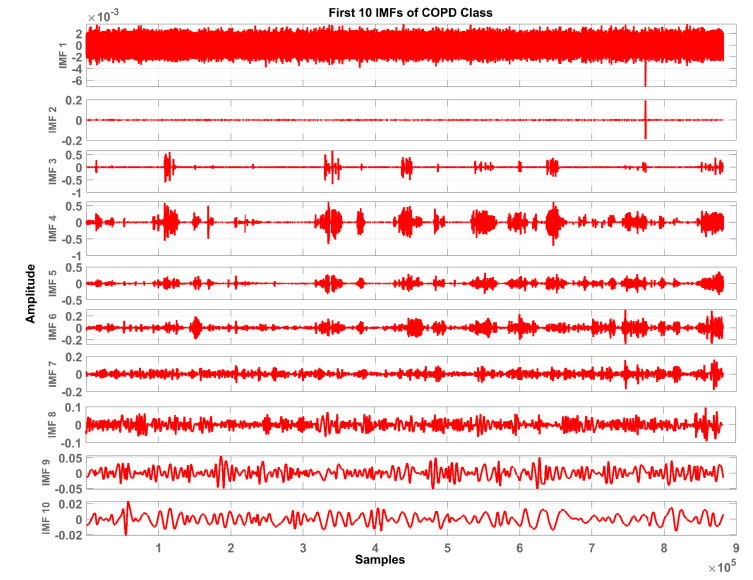
Graphical representation of IMF1-IMF10 after EMD analysis which corresponds to LS signal of COPD class.

**Figure 8:**
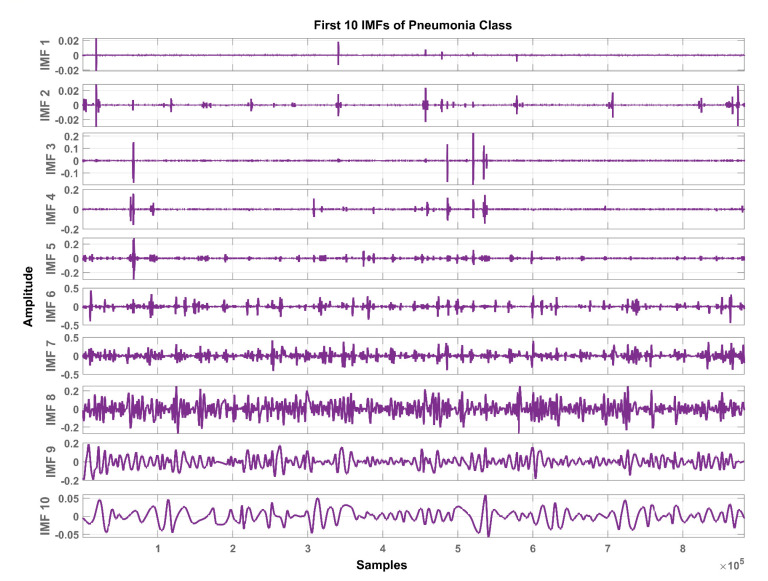
Graphical representation of IMF1-IMF10 after EMD analysis which corresponds to an LS signal of pneumonia class.

**Figure 9:**
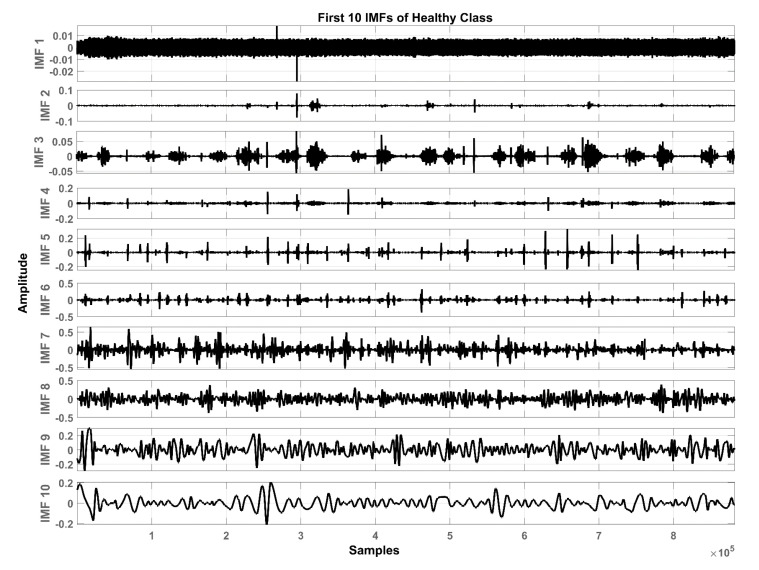
Graphical representation of IMF1-IMF10 after EMD analysis which corresponds to an LS signal of healthy class.

In total, ten IMFs are extracted and observed experimentally. Figure 7, Figure 8 and Figure 9 show the results of the COPD, pneumonia, and healthy class after implementation of the EMD technique. The EMD technique mines the signals associated with different intrinsic time scales in developing a collection of IMFs. Hence, we can localize any event in the time as well as the frequency domain. It is important to note that the frequency of the normal tracheal lung sound lies in the 60-600 Hz range [33]. IMF-2. IMF-3, IMF-4 is selected after experimentation.

The mean frequency ranges of the selected IMF in the case of COPD are IMF-2 (46.7 -412) Hz, IMF-3 (36.2-193) Hz, IMF-4 (29.5-142) Hz. In the case of pneumonia class, the mean frequency ranges include IMF-2 (476-5.22e+03) Hz, IMF-3 (10.6-144) Hz, and IMF-4 (8.89-114) Hz. The healthy subjects class comprises IMF-2 (179-5.7e+03) Hz, IMF-3 (139-1.05e+03) Hz and IMF-4 (12.9-427) Hz. Therefore, the selected ROI has a mean frequency range of 50 Hz to 5 kHz for all the classes in this study.

**Figure 10:**
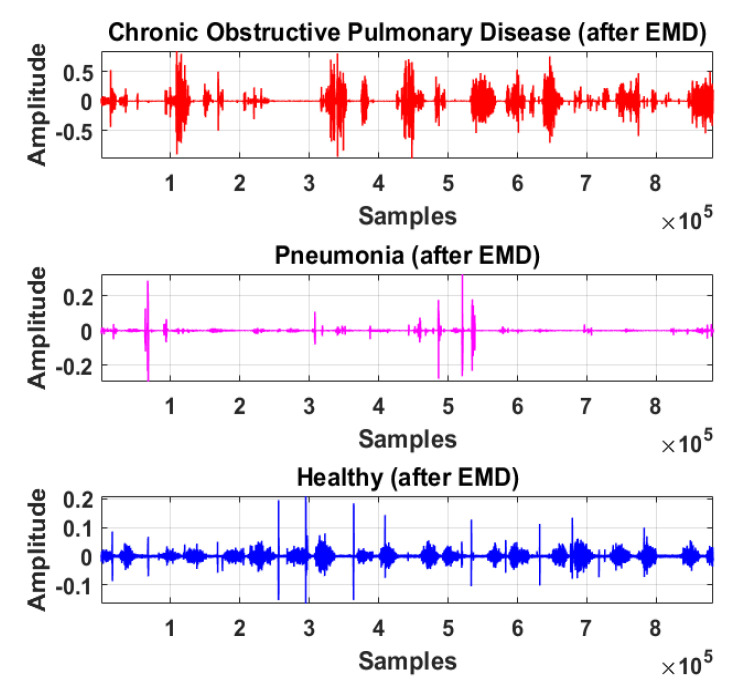
Time-domain graphical representation of reconstructed LS signal from IMF-2, IMF-3, and IMF-4 of COPD, pneumonia, and healthy class after EMD analysis.

**Figure 11:**
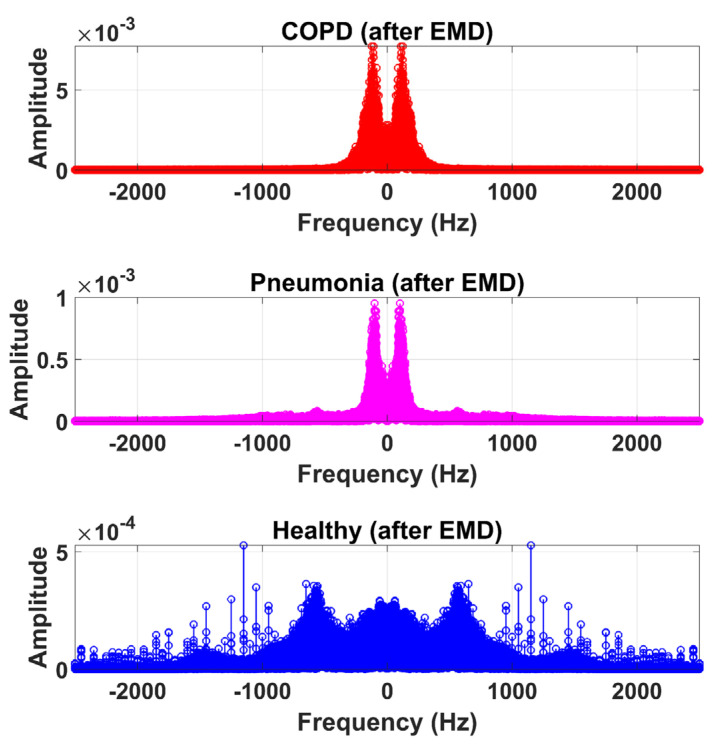
Frequency domain graphical representation of reconstructed LS signal from IMF-2, IMF-3, and IMF-4 of COPD, pneumonia, and healthy class after EMD analysis.

Therefore, LS information in IMF-2, IMF-3, and IMF-4 is the ROI in this research work. Higher IMFs were discarded due to the presence of noise and other lower-frequency components. LS signal is reconstructed by the selected IMF’s. The time and frequency domain representation of the LS signal reconstructed from IMF-2, IMF-3, and IMF-4 of raw LS signal is demonstrated in Figure 10 and Figure 11.

(ii) Discrete Wavelet Transform

**Figure 12:**
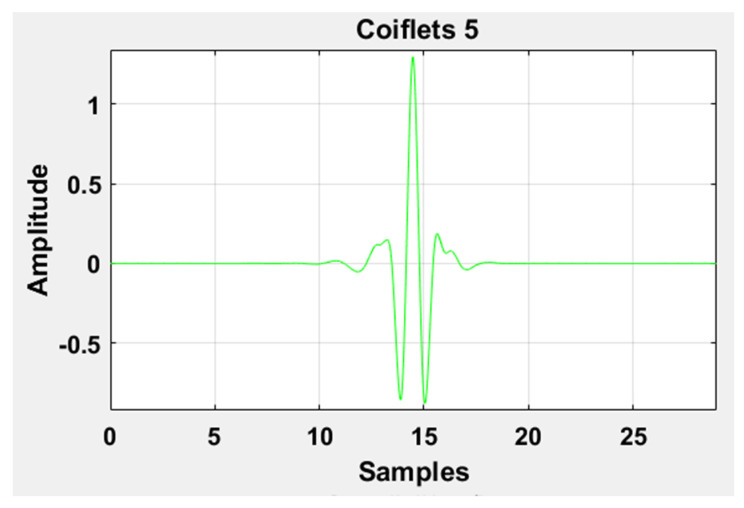
Graphical representation of the Coiflets 5 wavelet.

**Figure 13:**
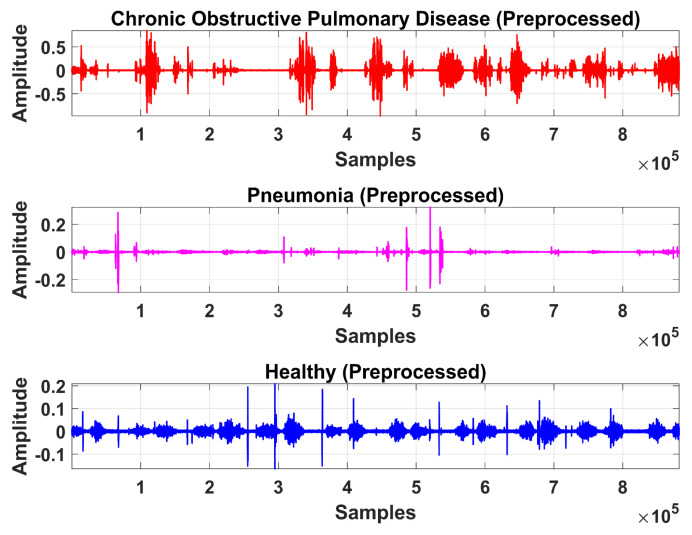
Time-domain graphical representation of preprocessed LS signal after denoising from DWT.

**Figure 14:**
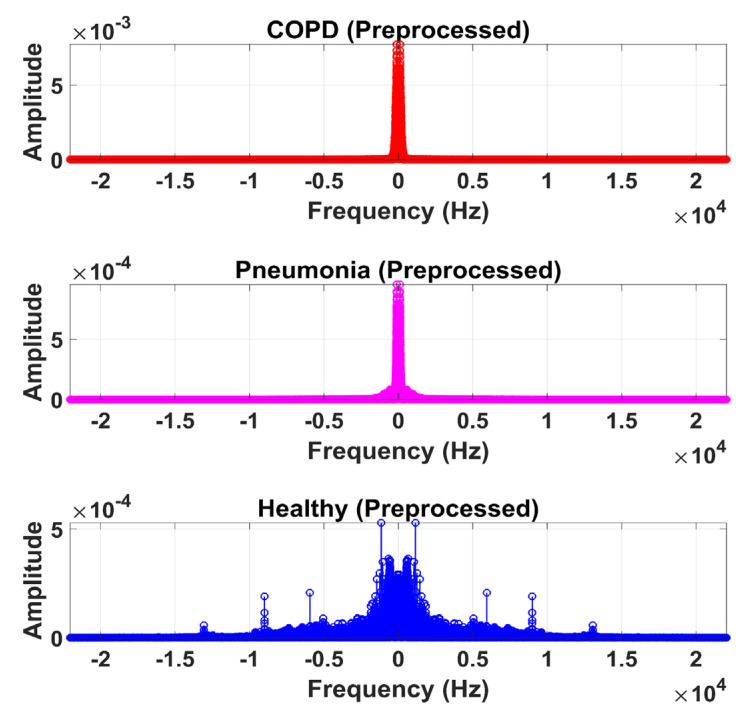
Frequency domain graphical representation of preprocessed LS signal after denoising from DWT.

Discrete wavelet decomposition (DWT) is a powerful tool to reduce noise by decomposing a signal. The signal L[n] obtained after the implementation of EMD is further denoised by the application of DWT via Equation (4):

(4) $$W_{L}\left[a,b\right]=1/\sqrt{\left|a\right|}\overset{}{\underset{R}{\sum }}L\left[n\right]\bar{\Phi \left[\frac{n-b}{a}\right]}$$

where a, b, Φ, and $W_{L}$ represents the scaling factor, translation factor, mother wavelet, and wavelet transformation function of the input time-series L[n], respectively. High-frequency noise can be added similarly as traces of heart sound (HS) become added during the data acquisition of LS. Therefore, DWT is used to ensure the removal of any irrelevant signal artifact from the ROI. According to the sampling frequency of the Nyquist sampling theorem, the sampling frequency of the experimental signal is set as 44.1 kHz. LS is decomposed into level 1 approximation (0 Hz ~ 11,025 Hz) and detail coefficients (11,025 Hz ~ 22,050 Hz) by the mother wavelet. The Coiflets5 wavelet [19] is used as a mother wavelet due to its morphological resemblance i.e. shape with LS signal to perform denoising. The Coiflets5 wavelet is shown in Figure 12. The approximation coefficients contain low frequencies whereas detailed coefficients contain high frequencies. Hard thresholding is applied to the detail coefficients. In the last step, the L[n] signal is reconstructed from approximation and thresholded detail coefficients. Finally, the processed signal is shown in Figure 13 and Figure 14 in the time and frequency domain, respectively. These figures demonstrate the LS signals with minimal presence of high-frequency noise and other irrelevant signal artifacts before the feature extraction stage.

## 3.3. Feature Extraction

Features are the major characteristics upon which a classifier distinguishes between the different LS classes. LS is non-stationary by nature. It is the reason that a single feature cannot forecast its nature. A total of 116 features were extracted which includes nineteen time-domain, 12 frequency-domain, 26 cepstral-domain, and 59 texture-based features. The texture features [34] are extracted from the spectrogram of signals (hop length: 10 samples, window length: 20 samples, window type: Hann window, overlap:10 samples). The summary of the extracted features is presented in Table 3.

The issue of unbalanced data is very common in the field of e-health. It refers to the presence of a huge number of data elements between the various classes. Some several methods or techniques can be used to replicate the data of the minority classes. The adaptive synthetic sampling method (ADASYN) is one of these augmentation data techniques which is used [35]. It helps to balance the data sample of normal and pneumonia subjects with COPD subjects using an appropriate number of samples.

## 3.4. Classification

Classification is performed by the classifier based on the distinct features extracted and selected in the previous stage. In this research, various classifiers were tested to observe the performance of the proposed method. These classifiers include decision tree (DT), linear discriminant (LD), logistic regression (LR), naïve Bayes-Gaussian (NB-G), naïve Bayes-kernel (NB-K), support vector machine- linear (SVM-L), support vector machine-quadratic (SVM-Q), support vector machine-cubic (SVM-C), support vector machine-fine Gaussian (SVM-FG), support vector machine-median Gaussian (SVM-MG), support vector machine-coarse Gaussian (SVM-CG), K nearest neighbor-fine (KNN-F), K nearest neighbor-medium (KNN-M), K nearest neighbor-coarse (KNN-Cor), K nearest neighbor- cosine (KNN-coss), K nearest neighbor-cubic (KNN-C), K nearest neighbor-weighted (KNN-W), ensemble boosted trees (Eboost), ensemble bagged trees (EBT), ensemble subspace discriminant (ESD), ensemble subspace KNN (ESKNN), and ensemble rUSBossted trees (ERT) on different time-domain (TD), frequency domain (FD), cepstral domain (CD) features and textural domain features i.e. local binary pattern (LBP).

QD classifiers consider that each class has its covariance matrix. Specifically, the predictor variables are not assumed to have common variance across each of the k levels in Y. Mathematically, it is supposed that observation from the *k*th class is of the form $X∼N\;\left(\mu_{k},\;\Sigma_{k}\right),$ where $\Sigma_{k}$ is a covariance matrix for the *k*th class. Under this supposition, the classifier assigns an observation to the class for which is the largest via Equation (5):

(5) $$\delta_{k}\left(l\right):=\mu_{k}^{⊤}\Sigma^{-1}l-\frac{1}{2}\mu_{k}^{⊤}\Sigma^{-1}\mu_{k}+\ln\left(\pi_{k}\right)$$

$\delta_{k}\left(l\right)$ is the estimated discriminant score that the observation will fall in the *k*th class within the response variable (i.e., default or not default) based on the value of the predictor variable *l*.

$\hat{\mu_{k}}$: the average of all the training observations from the *k*th class.

$\hat{\sigma_{2}}$: a weighted average of the sample variances for each of the K classes.

$\hat{\pi_{k}}$: the prior probability that an observation belongs to the *k*th class (not to be confused with the mathematical constant π≈3.14159).

Cross-validation is a resampling technique used to assess machine learning systems on a limited data set. Therefore, cross-validation of the system is performed on different folds. Accuracy (ACC), true positive (TPR), false-negative rate (FNR), positive predictive value (PPV), false discovery rate (FDR) are the main parameters on which system performance is measured using Equations (6)–(10):

(6) $$ACC=\frac{TP+TN}{TP+TN+FP+FN}$$

(7) $$TPR=\frac{TP}{TP+FN}$$

(8) $$FNR=\frac{FN}{FN+TP}$$

(9) $$PPV=\frac{FP}{FP+TP}$$

(10) FDR=1−PPV

In this research, cross-validation is performed on 5, 10, and 20 folds to demonstrate the system performance. Moreover, 20% hold out validation, and 25% hold out validation is also carried out to authenticate the outcome. All the results are generated using a Core i7 CPU 8 GB RAM, and 1 TB HDD with MATLAB R2018a.

## 4. Results and Discussion

**Figure 15:**
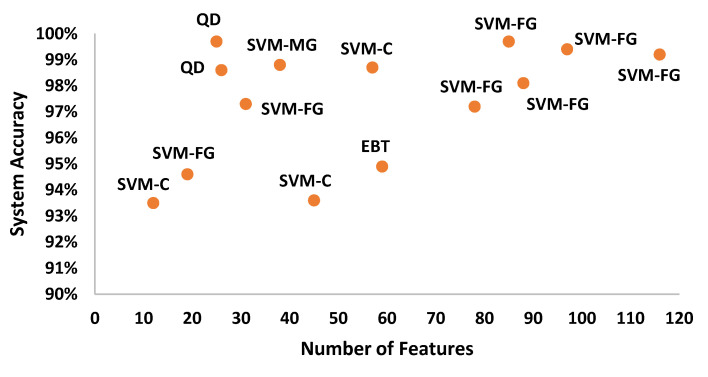
System accuracy of the classification techniques in comparison to different numbers of features.

**Figure 16:**
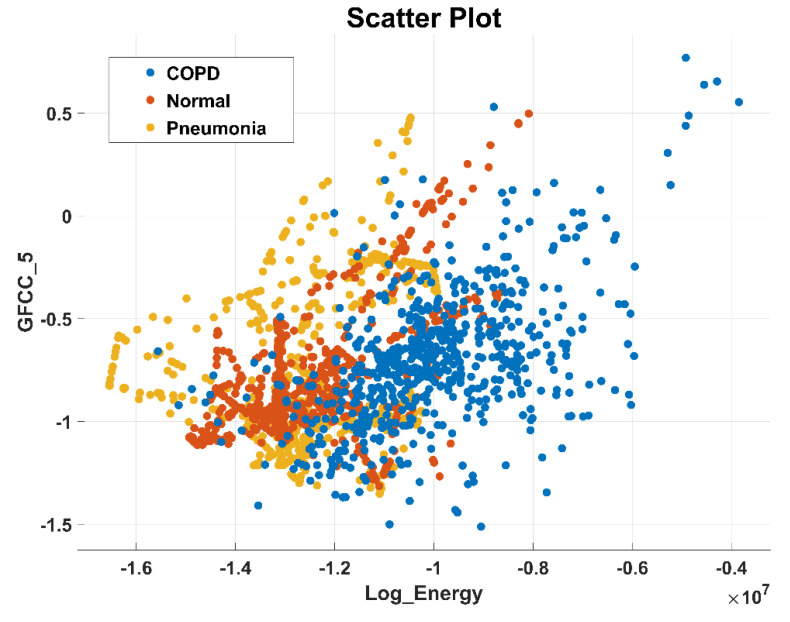
Scatter plot illustrating the minimum correlation between log energy (LE) and GFCC-5 feature of all classes.

**Figure 17:**
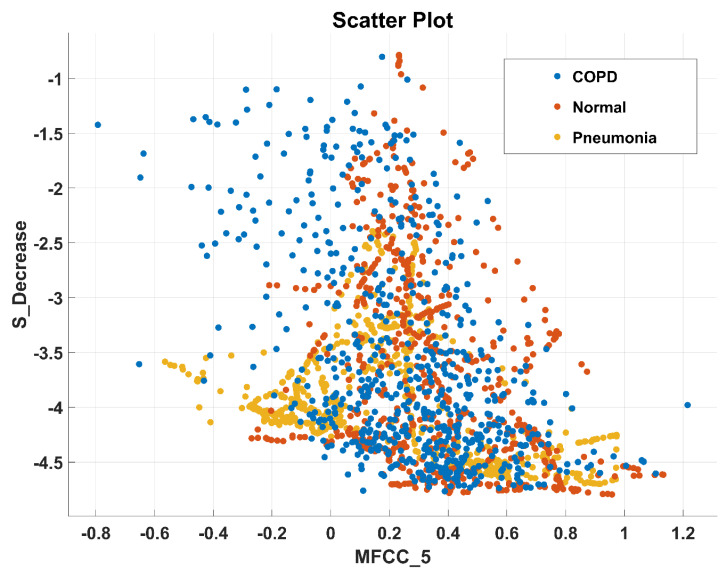
Scatter plot illustrating the minimum correlation between MFCC-10 and spectral decrease (SDec) features of all classes.

After the experimentation with different classifiers in this study, the quadratic discriminant (QD) classifier outperformed with the minimum number of features when compared with the performance of other classification techniques. Table 4 lists the different classifiers which have been tested on the proposed method with different combinations of features. Graphically, Figure 15 highlighted that the proposed technique has outperformed on SVM_FG classification method. It has achieved an accuracy of 99.70%, 99.40%, and 99.20% on different combinations of 85, 97, and 116 features, respectively. On the other hand, the QD classifier has provided a maximum accuracy of 98.60% with 26 features only. Investigation and selection of significant features to accomplish maximum accuracy with minimum features are important. It plays a key role to lower the burden of the computational cost of those features which are not paying a significant role at the classification stage. As the proposed system is simulated to develop a stand-alone embedded system to identify the pulmonary abnormalities in the future so it will help to enhance the robustness of the system. The feature significance is characterized by analyzing the scatter plot and statistics of different features presented in Appendix A. Figure 16 and Figure 17 demonstrate the scatter plot between log energy, and GFCC-5, MFCC-10, and spectral decrease of all classes. In Figure 16, it can be visualized that there exists a minimum correlation between LE and GFCC-5 features of focused LS data. The minimum correlation also reflects the large spread among feature estimates, so a classifier can perform well with features that have less correlation and more spread between them. The same can be observed in Figure 17 regarding MFCC-10 and SDec estimations of focused data. Therefore, it is expected that such features will play a vital role in the classification when fed to the classifier. The significance of other features is analyzed experimentally in the same fashion.

Table 5 lists the significant features that are selected for the classification of pulmonary pathologies after observing their scatter plots. Experimentation on the system’s implementation with different classifiers proved the optimum performance of the QD technique. The system achieved 99.7% accuracy with the QD classifier when implemented with 25 features only. It accomplished the achievement of 78.44% feature reduction by using the backward elimination method [36] to reduce the time complexity and unnecessary computation. The mathematical description of the substantial features is provided in Table 6. These features have provided an essential basis for the classification of COPD, pneumonia, and healthy LS. Standard deviation (SD) in the time domain provides the signal information about its spread from the mean. In the frequency domain, spectral standard deviation (SSD) provides the spread of signal frequencies from its mean. Variation in signal strength in COPD and pneumonia LS is compared with healthy LS class by the log energy (LE). The peak to peak (PP) value returns the highest and lowest value difference in the LS signal [24]. Spectral skewness (SSkw) estimates the distribution symmetry of the spectral magnitude about their arithmetic mean. It specifies the extent of non-similarities among spectral magnitudes. It has a low value for flat and a high value for the vibrational spectrum. The spectral kurtosis (SK) estimates the resemblance of spectral magnitude distribution with a gaussian distribution. Its main application is to figure out the occurrence of peaks in the LS signal spectrum. The high value of spectral kurtosis is due to high peaks in the spectrum of an LS segment. Spectral roll-off (SRO) quantifies the spectrum concentration. The spectral decrease (Sdec) calculates that how steeper spectral envelope decrease over frequency. The spectral flux (SF) estimates the variation in the shape of the spectrum by calculating the average difference among consecutive STFT frames [16]. MFCC and GFCC belong to the cepstral features class. We have adopted to use it in LS pathologies motivated by its performance in speech recognition and its robust nature in noise reduction [37].

**Figure 18:**
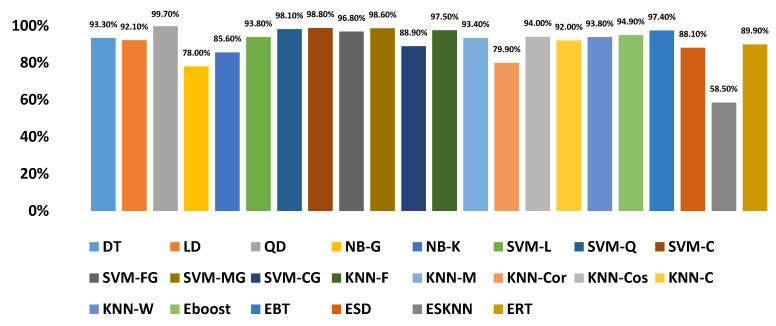
Accuracy of the proposed diagnosis method with an optimized number of features on different classifiers.

Graphically, the system accuracy on different classifiers with selected feature sets is demonstrated in Figure 18. It demonstrates that QD showed 99.70% accuracy upon selected features as compared to any other classifier when denoising is performed by WT.

**Figure 19:**
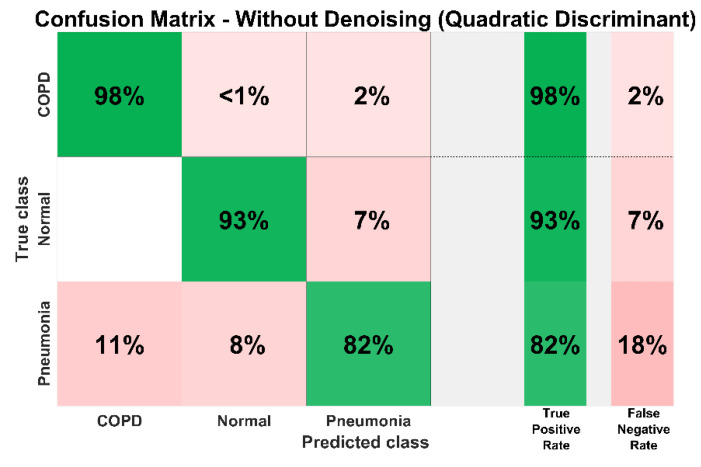
System confusion matrix (QD classifier) before denoising.

Figure 19 shows the confusion matrix of the proposed diagnosis methodology for the identification of COPD, pneumonia, and healthy LS without denoising. It demonstrates the classification performance of the proposed system for all classes. It can be observed that without denoising the true positive rate of COPD, pneumonia, and healthy LS diagnosis are 98%, 93%, and 82% respectively. In the case of pneumonia, 11%, and 8%, samples are falsely predicted as COPD and healthy class respectively overall depicts an 18% false-negative rate. In the normal or healthy class, 0% of samples were wrongly predicted as COPD whereas 7% of healthy samples are classified as pneumonia, equivalent to a 7% false-negative rate. In the COPD class, <1% of samples are wrongly identified as normal while 2% of COPD samples are falsely identified as pneumonia cases.

**Figure 20:**
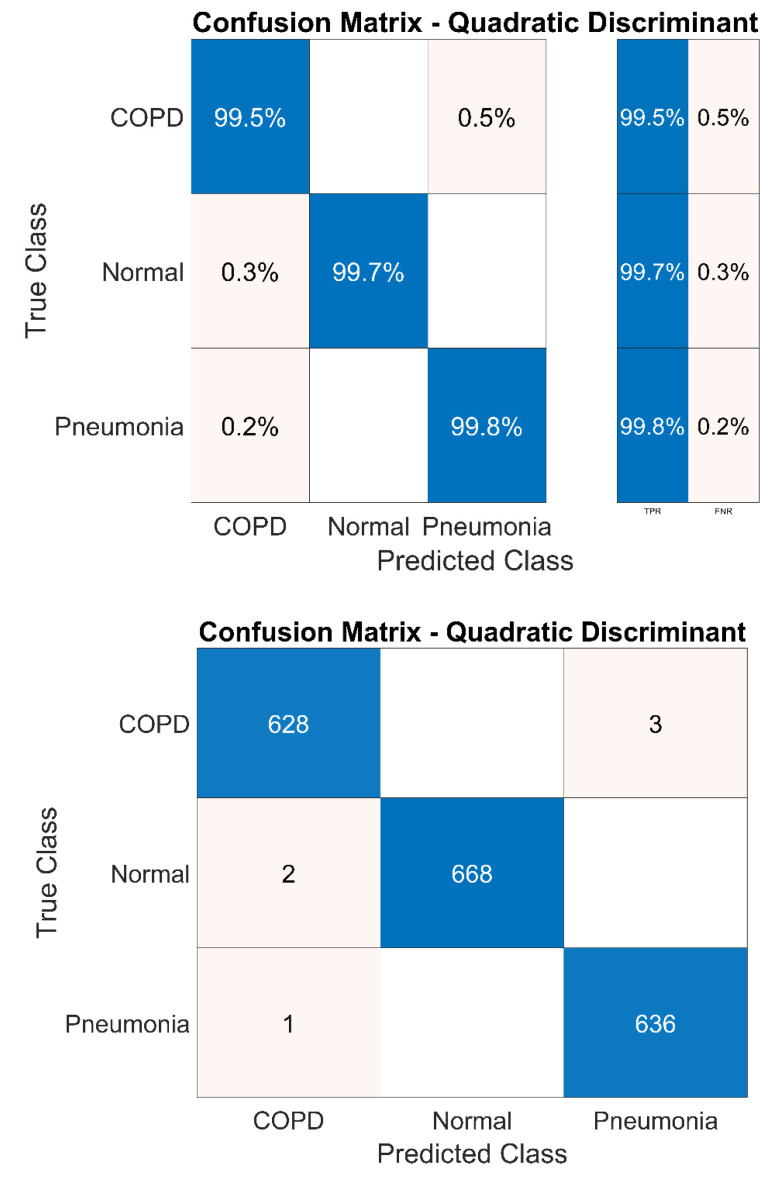
System confusion matrix (QD classifier) after denoising.

Experimentation performed with the DWT denoising technique after selecting the ROI via EMD technique has improved the true positive rate of all classes to greater than 99%. This is demonstrated in Figure 20. It can be seen that from a total of 631 COPD samples, only three samples are falsely identified. Similarly, two normal and one pneumonia samples are wrongly identified by the system from the overall 670 and 636 samples, respectively.

5, 10, 15, and 20 fold cross-validation is performed to authenticate the system performance which can be observed in Table 7. Variation in folds for cross-validation has not affected the system’s robustness. Almost the same accuracy has been achieved in each case. The hold-out technique is also performed to validate system performance. The system achieved 99.70% and 99.80% when the 20% and 25% hold out validation method is implemented. In the 20% hold-out method, data is divided into two portions for training and testing i.e. 80% and 20%. 80% of the data is used for training whereas 20% of data is used for testing. this gives a better indication of how well the proposed method performs on unseen data.

Different studies have been performed on the classification of pulmonary diseases. The proposed technique has outperformed in comparison with various published techniques to identify the pulmonary abnormalities from LS analysis in terms of the number of features, accuracy sensitivity, and specificity of the method.

According to the best of our knowledge, there is a single previous research work on COPD and pneumonia LS, but the main aim of that study was to analyze the acoustic parameters from respiratory signals in COPD and pneumonia patients. These parameters were not further utilized by the researcher to classify the COPD and pneumonia LS [20]. Nevertheless, the number of extracted features in some existing research works is less than the proposed technique but it can only identify a single pulmonary illness from LS analysis [6,12,18,25,26]. Details are presented in Table 8.

Regarding the comparative study and performance evaluation, there should be similar methods, but even the most recent literature mainly focused on COPD, pneumonia, and healthy LS identification is limited. That’s the reason the comparison of this research work with similar methods is difficult, although we believe this research represents an innovative step towards screening of COPD, pneumonia, and healthy LS. The proposed technique has provided an efficient approach with outstanding classification accuracy. It has outperformed as compared to existing techniques on other multiple pulmonic pathologies from LS analysis due to its simple statistical features, low computation, and accuracy [6,7,12,20,22,24,25,28,29]. The performance analysis of the proposed diagnostic technique with existing pulmonic pathologies methods is shown in Table 9. As mentioned, these techniques focused on different combinations of lung disease and there is a lack of research in which LS classification is performed for COPD and pneumonia identification only. Therefore, the presented research work is a progressive and fruitful initiative to explore the use of LS for the identification of normal, COPD, and pneumonia subjects.

## 5. Conclusions

In this article, an efficient method is proposed for the classification of pulmonary pathologies from LS analysis. It provides a non-invasive, convenient to use, and low-cost solution for diagnosis of COPD and pneumonia issues which are one of the leading causes of death worldwide. The research work is based on the ICBHI open access LS database which endorses the authenticity of the focused data in the development of the proposed method. ROI extracted through EMD and further denoising via DWT has proven the best approach to unfold the main information required for feature extraction, verified by the statistical analysis of extracted features. Feature reduction through the back elimination method and experimentation on different feature sets using various classification techniques has established the best performance of the quadratic discriminant classifier with 25 features only. The proposed study has performed classification with 99.7% accuracy, TPR>99%, and FNR<1%. The system cross-validation result on different folds as well as hold out validation method substantiates the reported accuracy. The use of simple statistical features also ensures the minimal computational cost of presented research work. The proposed method is purely based on automated LS analysis. No clinical information is needed like other techniques based on knowledge graphs. Although various studies have been found on different lung diseases, to the best of our knowledge, we have found a lack of research targeting particularly the solution for COPD and pneumonia issues via signal processing and machine learning approach. Therefore, the proposed research work is a progressive and innovative approach to the diagnosis of COPD and pneumonia. The proposed method will help to monitor pulmonary health in COPD and pneumonia patients. It is ready to embed approach due to the simple technique. Furthermore, it is a promising method to assist pulmonologists as a counterpart to their clinical diagnosis. Implementation of the proposed technique on specific hardware and designing a stand-alone portable system for pulmocare can be done to bring this research one step ahead. The performance analysis of the presented research work needs authentication on self-collected data.

## Figures and Tables

**Table 1:** Recording Equipment Used to develop Lung Sound Database.

Sr. No	Equipment
1.	Welch Allyn Meditron Master Elite Plus Stethoscope Model 5079-400
2.	3M Littmann 3200
3.	3M Littmann Classic II SE

**Table 2:** Demographic Information of Focused LS Database.

Description	Detail
Total number of LS signal sets (COPD, pneumonia, healthy)	703
The sampling frequency of recording equipment	44.1 kHz
Bits/ sample	16
Average recording duration	21.5s
Number of participants	57 (50 adults & 7 children)
Gender	40 males, 17 females
Age	Adults: 69.44 ± 8.24 Years, Children: 6.06 ± 5.98 Years

**Table 3:** List of extracted features for LS analysis.

Time Domain Features (19)	Spectral (S) Domain Features (12)	Cepstral Features (26)	Texture Features (59)
Mean, St. Deviation, Skewness, Kurtosis, Peak to Peak, Root Mean Square, Crest Factor, Shape Factor, Impulse Factor, Margin Factor, Energy, Peak to RMS, Root Sum of Squares, Shannon Energy, Log Energy, Mean Abs Deviation, Median Abs Deviation, Average Frequency, Jitter	S.Mean, S.St. Deviation, S.Skewness, S.Kurtosis, S.Centriod, S.Flux, S.Rolloff, S.Flateness, S.Crest, S.Decrease, S.Slope, S.Spread	MFCC, GCC	LBP

MFCC: Mel Frequency Cepstral Coefficient, GCC: Gammatone Cepstral Coefficients, LBP: Local Binary Patterns. (Appendix A lists the statistics of all features in each class).

**Table 4:** Accuracy of different classifiers on various feature groups.

	FEATURE GROUPS													
CLASSIFIERS		TD	FD	CD	Texture	TD+FD	TD+CD	FD+CD	CD+Texture	TD+Texture	TD+FD+CD	TD+FD+Texture	FD+CD+Texture	TD+FD+CD+Texture
	DT	87.30%	86.80%	94.50%	92.50%	89.50%	95.40%	94.60%	94.60%	93.70%	95.00%	94.20%	94.60%	95.00%
	LD	76.40%	76.50%	91.10%	73.90%	82.70%	93.40%	93.60%	92.00%	-	94.20%	-	93.70%	-
	QD	-	84.70%	98.60%	-	-	-	-	-	-	-	-	-	-
	LR	-	-	-	-	-	-	-	-	-	-	-	-	-
	NB-G	59.30%	68.00%	82.40%	-	69.20%	73.40%	79.80%	-	-	75.10%	-	-	-
	NB-K	66.80%	71.10%	88.30%	41.70%	73.00%	85.50%	88.90%	47.10%	43.50%	85.90%	44.70%	48.00%	48.50%
	SVM-L	79.30%	80.10%	94.90%	83.90%	85.30%	96.00%	95.30%	95.50%	91.30%	96.00%	91.90%	96.10%	95.90%
	SVM-Q	91.00%	88.70%	97.90%	92.10%	94.40%	98.10%	98.30%	97.80%	95.60%	98.10%	96.00%	97.90%	98.00%
	SVM-C	93.60%	93.50%	98.50%	58.00%	97.00%	98.70%	98.50%	96.00%	88.00%	98.60%	94.20%	98.60%	98.60%
	SVM-FG	94.60%	91.30%	96.20%	91.40%	97.30%	97.20%	95.80%	99.70%	97.20%	97.20%	98.10%	99.40%	99.20%
	SVM-MG	85.60%	82.90%	98.40%	79.60%	91.40%	98.60%	98.80%	97.10%	90.40%	98.70%	92.20%	97.80%	97.80%
	SVM-CG	70.00%	75.40%	90.80%	61.40%	79.70%	94.00%	93.10%	85.70%	73.50%	93.20%	78.70%	89.90%	90.70%
	KNN-F	92.70%	91.60%	97.90%	93.80%	95.00%	97.50%	97.50%	98.00%	94.30%	97.00%	95.50%	97.80%	97.00%
	KNN-M	87.50%	84.90%	94.10%	86.70%	89.40%	94.00%	92.60%	94.30%	90.00%	92.90%	89.80%	93.20%	93.00%
	KNN-Cor	65.80%	71.40%	80.60%	71.30%	74.80%	84.10%	81.00%	81.80%	70.30%	84.20%	76.30%	81.30%	87.70%
	KNN-Cos	88.10%	86.00%	94.90%	87.50%	90.20%	94.60%	94.60%	95.20%	90.90%	94.40%	91.00%	94.30%	94.80%
	KNN-C	87.90%	85.00%	93.80%	86.60%	88.80%	93.60%	92.40%	94.30%	89.90%	92.00%	89.50%	92.90%	92.40%
	KNN-W	89.60%	88.40%	9.60%	90.10%	91.10%	94.20%	93.30%	94.50%	91.40%	93.30%	91.00%	93.70%	93.20%
	Eboost	84.80%	83%	96.40%	89.30%	90.50%	96.60%	96.60%	96.60%	94.70%	96.30%	94.50%	96.70%	96.70%
	EBT	93.80%	91.50%	97.10%	94.90%	95.70%	97.30%	96.90%	97.90%	96.30%	97.20%	97.00%	97.50%	97.60%
	ESD	71.70%	72.30%	88.70%	71.40%	79.50%	92.30%	91.60%	90.00%	81.40%	93.10%	85.30%	93.30%	94.00%
	ESKNN	68.70%	77.30%	97.50%	92.00%	74.90%	69.30%	83.40%	97.70%	69.00%	74.60%	75.40%	84.20%	74.70%
	ERT	77.00%	78.80%	91.20%	84.40%	84.20%	92.40%	92.90%	92.40%	88.40%	92.80%	88.30%	92.80%	93.00%

**Table 5:** List of Selected Features Extracted and Performance evaluation.

Selected Features from Time, Frequency, and Cepstral Domain	Classifier	Performance Outcome
**Time** (Standard Deviation, Peak to Peak, Log Energy), **Spectral** (Spectral Standard Deviation, Spectral Skewness, Spectral Kurtosis, Spectral Flux, Spectral Roll Off, Spectral Decrease), **Cepstral** (MFCC (3-10), GFCC (3-10))	Quadratic discriminant	Overall Accuracy 99.70%

**Table 6:** Mathematical Description of the selected features for classification of LS signal L[n].

Feature	Mathematical Representation
Standard Deviation (SD)	$SD=\sqrt{\frac{\sum_{i=1}^{N}{\left(Li-L_{Mean}\right)}^{2}}{n}}$ where L:L[n]
Peak to Peak (PP)	$PP=L_{max}-L_{min}$ Where $L_{max}$ and $L_{min}$ is the minimum and maximum value in the time domain
Log Energy (LE)	$LE=\log\left[\sum_{i=1}^{N}{\left(\left|Li\right|\right)}^{2}\right]$
Spectral Standard Deviation (SSD)	$\sigma =\sqrt{\frac{\sum_{i=1}^{N}{\left(Li-\left|L_{Mean}\right|\right)}^{2}}{n}}$ where L:L[ω]
Spectral Skewness (SSkw)	$SSkew=\frac{2}{n}\left[\frac{\sum_{i=1}^{n/2-1}{\left(\left|L_{i}\right|-\left|L_{Mean}\right|\right)}^{3}}{\sigma^{3}}\right]$ where L:L[ω]
Spectral Kurtosis(SK)	$SK=\frac{2}{n}\left[\frac{\sum_{i=1}^{n/2-1}{\left(\left|L_{i}\right|-\left|L_{Mean}\right|\right)}^{2}}{\sigma^{4}}\right]-3$ where L:L[ω]
Spectral Flux (SF)	$SK=\frac{2}{i}\sqrt{\sum_{i=0}^{i/2-1}{\left(\left|L_{n,i}\right|-\left|L_{n,\;i-1}\right|\right)}^{2}}$
Spectral Roll Off (SRO)	If $m_{th}$ DFT coefficient corresponds to the spectral roll-off of the $k_{th}$ frame, then $\sum_{i=1}^{n}L_{k}\left(i\right)=C\sum_{i=1}^{F_{L}}L_{k}\left(i\right)$ C is the adapted percentage: 95% and L: L[ω]
Spectral Decrease (SDec)	$SDec=\frac{\sum_{i=1}^{i/2-1}\frac{1}{i}{\left(\left|L_{i}\right|-\left|L_{0}\right|\right)}^{}}{\sum_{i=1}^{1/2-1}\left|L_{i}\right|}$ where L:L[ω]
Mel frequency cepstral coefficient (MFCC)	In MFCC, (i) Frame blocking or windowing to get 50 to 60ms. (ii) Performing a discrete Fourier transform (iii) computing logarithm of the signal. (iv) Deforming the frequencies on a Mel scale, followed by applying the discrete cosine transform (DCT). Mel scale is calculated as follows: $Mel\;Scale=2595\;log_{10}\left(1+\frac{f}{700}\right)$ ‘f’ refers to frequency ranges from 0 to fs.
Gammatone Frequency Cepstral Coefficient (GFCC)	In GCC, (i) Firstly, the signal is passed through gammatone filter bank which consists of 64 Channels. (ii) Take the absolute value at each channel and reduce it to 100 Hz as a way of time windowing. (iii) Take cubic root on the time-frequency representation. (iv) Deforming the frequencies on an equivalent rectangular bandwidth (ERB) scale Apply DCT to derive cepstral features. ERB scale is calculated as follows. $ERB=Alog_{10}\;\left(1+hz\left(0.00437\right)\right)$ where $A\;=\;\frac{1000log_{e}\left(10\right)}{\left(24.7\right)\left(4.37\right)}$ ‘hz’ refers to frequency ranges i.e. 0-f_s_.

**Table 7:** Cross-validation of the proposed method with different folds.

Evaluation	Classes	ACC %	TPR %	FNR %	PPV %	FDR %
(5, 10, 15, and 20) Fold Cross-Validation	COPD	99.6	>99	<1	99	1
	Normal		>99	<1	100	0
	Pneumonia		>99	<1	>99	<1
20% Hold Out Validation	COPD	99.7	99	1	100	0
	Normal		100	0	100	0
	Pneumonia		100	0	99	1
25% Hold Out Validation	COPD	99.8	100	0	99	1
	Normal		99	1	100	0
	Pneumonia		100	0	100	0

ACC: Mean accuracy, TPR: True Positive, FNR: False Negative Rate, PPV: Positive Predictive Value, FDR: False Discovery Rate.

**Table 8:** Comparative analysis of the purposed technique with similar lung pathology methods.

Class	Number of Features	Accuracy (%)
Pneumonia [12]	13	87.87
Pneumonia [6]	18	90.06
Pneumonia [18]	7	99.70
COPD [25]	25	85.10
COPD [26]	27	95.10
COPD, pneumonia (This method)	25	99.70

**Table 9:** Performance analysis of the proposed diagnosis methodology for COPD and pneumonia identification with current techniques on lung pathologies.

Classes	Method	Results (%)
Crackles, Crackles+ Wheeze, Normal, Wheeze [5]	STFT, WT, SVM	ACC: 49.86
Normal, Pneumonia [6]	SA	ACC: 91.98 SEN:92.06 SPE: 90.68
Pneumonia and Asthma [7]	NN	SEN: 89, SPE:100
Normal, Pneumonia [12]	WT, LR ANN	SEN: 94 SPE:63
Normal, Pneumonia [18]	EMD, KNN	ACC: 99.7
Normal, COPD and Pneumonia [20]	SA	-
Normal Asthma and COPD [22]	ANN	ACC:60.33 SEN: 65 SPE:54.2
Normal, Asthma, Bronchitis [24]	EMD, KNN	ACC: 99.3
COPD [25]	KT	ACC: 85.1
COPD [26]	KG, ML	ACC:95.1
COPD, Healthy, Pneumonia, Asthma, Bronchiectasis, Bronchiolitis [29]	CNN	SEN: 98.8 SPE:98.6
Crackles, Crackles+ Wheeze, Normal, Wheeze [28]	CNN	ACC i: 65.5 ii: 63.09
Normal, COPD, Pneumonia (This Method)	EMD, WT, QD	ACC: 99.8%

Knowledge graph: KG, Short-time Fourier Transform: STFT, Linear regression: LR, Statistical analysis: SA, Neural network: NN, Knowledge transfer: KT, ACC: Mean accuracy. SEN: Sensitivity, SPE: Specificity.

## Appendix A. Feature Statistics of All Classes

**Table A1:** Feature Statistics of All Classes.

Features	COPD	Pneumonia	Normal
Mean	$-1.72656\;\times \;10^{-5}\pm 7.0\;\times \;\;10^{-4}$	$-2.03\;\times \;10^{-6}\pm 8.79\;\times \;10^{-5}$	$-1.63359\;\times \;10^{-4}\pm 7.0\;\times \;\;10^{-4}$
Standard Deviation	$5.163751\;\times \;10^{-2}\pm 5.13\;\times \;10^{-2}\;$	$1.49\;\times \;10^{-2}\pm 1.02\;\times \;10^{-2}$	$2.482\;\times \;10^{-2}\pm 2.2773\;\times \;10^{-2}$
Skewness	$-1.5\;\times \;10^{-2}\pm 2.62$	$1.5910^{-1}\pm 1.61$	$4.7830\;\times \;10^{-1}\pm 1.9630$
Kurtosis	134.169±2241.2391	$1.59\;\times \;10^{2}\pm 1.52\;\times \;10^{2}$	1.59±2.433818
Peak_to_Peak	$1.410\pm 9.02\;\times \;10^{-1}\;$	$6.01\;\times \;10^{-1}\pm 3.55\;\times \;10^{-1}$	$7.15\;\times \;10^{-1}\pm 3.725\;\times \;10^{-1}$
Root_Mean_Square	$5.164\;\times \;10^{-2}\pm 5.14\;\;\times \;\;10^{-2}$	$5.164\;\times \;10^{-2}\pm 5.14\;\times \;10^{-2}$	$2.482\;\times \;10^{-2}\pm 2.278\;\times \;10^{-2}$
Crest_Factor	19.138±10.612	$-2.03\;\times \;10^{-6}\pm 8.79\;\times \;10^{-5}$	23.196±15.770
Shape_Factor	2.837±1.034	$1.49\;\times \;10^{-2}\pm 1.02\;\times \;10^{-2}$	3.557±0.984
Impulse_Factor	58.137±51.088	$1.59\;\times \;10^{-1}\pm 1.61$	89.250±80.107
Margin_Factor	13082.53±35130.948	$1.59\;\times \;10^{2}\pm 1.52\;\times \;10^{2}$	49152.215±79027.13
Energy	4676.043±9996.639	$6.01\;\times \;10^{-1}\pm 3.55\;\times \;10^{-1}$	1085.5845±2096.891
Peak_to_Root_Mean_Square	20.731±11.642	$1.49\;\times \;10^{-2}\pm 1.02\;\times \;10^{-2}$	24.585±16.806
Root_Sum_of_Squares	48.498±48.246	$2.19\;\times \;10^{1}\pm 6.33$	23.310±21.399
Shannon_Energy	7387.506±11252.795	$3.39\pm 9.47\;\times \;10^{-1}$	3021.558±4586.466
Log_Energy	−10006818.89±1718189.627	$7.87\;\times \;10^{1}\pm 4.21\;\times \;10^{-1}$	−12431065.34±1398211.96
Mean_Absolute_Deviation	$2.104\;\times \;10^{-2}\pm 2.542\;\times \;10^{-2}$	$4.09\;\times \;10^{4}\pm 5.02\;\times \;10^{4}$	$8.181\;\times \;10^{-3}\pm 9.453\;\times \;10^{-3}$
Median_Absolute_Deviation	$6.718\;\times \;10^{-3}\pm 1.216\;\times \;10^{-2}$	$2.97\;\times \;10^{2}\pm 4.25\;\times \;10^{2}$	$1.449\;\times \;10^{-3}\pm 1.947\;\times \;10^{-3}$
Average_Frequency	$4.643\;\times \;10^{-3}\pm 1.067\;\times \;10^{-2}$	$2.40\;\times \;10^{1}\pm 8.09$	$5.4196\;\times \;10^{-3}\pm 1.252\;\times \;10^{-2}$
Jitter	14560.686±35699.968	$1.40\;\times \;10^{1}\pm 9.60$	16136.403±39412.294
Spectral Mean	624.722±1123.544	$1.21\;\times \;10^{3}\pm 1.45\;\times \;10^{3}$	1035.002±1421.975
Spectral Std_Deviation	990.575±1590.132	$-1.26\;\times \;10^{7}\pm 1.38\;\times \;10^{6}$	1195.782±1506.922
Spectral Skewness	3.3043±2.877	$5.22\;\times \;10^{-3}\pm 4.45\;\times \;10^{3}$	2.378±1.664
Spectral Kurtosis	23.602±37.359	$1.19\;\times \;10^{-3}\pm 1.03\;\times \;10^{-3}$	12.437±14.021
Spectral Centriod	458.586±119.633	$6.93\;\times \;10^{-4}\pm 7.92\;\times \;10^{-4}$	451.540±101.3461
Spectral Flux	$1.666\;\times \;10^{-3}\pm 0.001.216\;\times \;10^{-3}$	$1.70\;\times \;10^{4}\pm 2.36\;\times \;10^{4}$	$7.20957\;\times \;10^{-4}\pm 3.6273\;\times \;10^{-4}$
Spectral Rolloff	4769.686±892.684	$3.78\;\times \;10^{2}\pm 6.97\;\times \;10^{2}$	4874.620±1002.519
Spectral Flateness	$4.637\;\times \;10^{-2}\pm 4.340\;\times \;10^{-2}$	$6.71\;\times \;10^{2}\pm 1.22\;\times \;10^{3}$	$4.4622\;\times \;10^{-2}\pm 4.339\;\times \;10^{-2}$
Spectral Crest	$5.88\;\times \;10^{-1}\pm 6.694\;\times \;10^{-2}$	4.16±1.99	$5.896\;\times \;10^{-1}\pm 6.69\;\times \;10^{-2}$
Spectral Decrease	$-3.560\pm 9.69\;\times \;10^{-1}$	$2.63\;\times \;10^{1}\pm 1.82\;\times \;10^{1}$	−3.6488±0.9344
Spectral Slope	$-8.811\;\times \;10^{-3}\pm 8.338\;\times \;10^{-3}$	$4.13\;\times \;10^{2}\pm 1.12\;\times \;10^{1}$	$-5.181\;\times \;10^{-3}\pm 4.0841\;\times \;10^{-3}$
Spectral Spread	842.519±79.572	$5.27\;\times \;10^{-4}\pm 2.89\;\times \;10^{-4}$	836.339±65.276
MFCC_1	11.903±2.190	$4.41\;\times \;10^{3}\pm 4.59\;\times \;10^{2}$	8.7944±1.7834
MFCC_2	3.335±1.180	$2.70\;\times \;10^{-2}\pm 1.71\;\times \;10^{-2}$	3.4474±1.0772
MFCC_3	$5.382\;\times \;10^{-1}\pm 6.749\;\times \;10^{-1}$	$6.15\;\times \;10^{-1}\pm 3.77\;\times \;10^{-2}$	0.4647±0.5036
MFCC_4	0.552367871±0.384043217	$-3.95\pm 5.64\;\times \;10^{-1}$	0.3742±0.3563
MFCC_5	0.25597491±0.28060119	$-2.89\;\times \;10^{-3}\pm 1.62\;\times \;10^{-3}$	0.3547±0.2468
MFCC_6	0.331806541±0.201000215	$8.11\;\times \;10^{2}\pm 8.25$	0.40014±0.1958
MFCC_7	0.117074904±0.189924798	7.56±1.68	0.03602±0.1994
MFCC_8	0.133825725±0.176784491	$3.61\pm 9.24\;\times \;10^{-1}$	$-2.59993\;\times \;10^{-3}\pm 0.1555$
MFCC_9	0.084564833±0.143536172	$9.22\;\times \;10^{-1}\pm 4.78\;\times \;10^{-1}$	$8.5559\;\times \;10^{-2}\pm 9.6075\;\times \;10^{-2}$
MFCC_10	0.125658833±0.1157861	$6.51\;\times \;10^{-1}\pm 4.04\;\times \;10^{-1}$	0.157698±0.0956
MFCC_11	$5.238\;\times \;10^{-2}\pm 9.380\;\times \;10^{-2}$	$2.29\;\times \;10^{-1}\pm 3.40\;\times \;10^{-1}$	$-9.523\;\times \;10^{-3}\pm 7.8486\;\times \;10^{-2}$
MFCC_12	$6.680\;\times \;10^{-2}\pm 9.548\;\times \;10^{-2}$	$2.88\;\times \;10^{-1}\pm 2.54\;\times \;10^{-1}$	$1.3285\;\times \;10^{-2}\pm 6.8225\;\times \;10^{-2}$
MFCC_13	$4.071\;\times \;10^{-2}\pm 8.991\;\times \;10^{-2}$	$9.31\;\times \;10^{-2}\pm 1.90\;\times \;10^{-1}$	$6.2124\;\times \;10^{-2}\pm 5.60953\;\times \;10^{-2}$
GFCC_1	−12.543±2.100	$1.64\;\times \;10^{-1}\pm 1.31\;\times \;10^{-1}$	−16.1741±1.4561
GFCC_2	4.906±1.232	$1.19\;\times \;10^{-1}\pm 7.72\;\times \;10^{-2}$	4.3728±1.289
GFCC_3	−1.098±1.046	$1.53\;\times \;10^{-1}\pm 6.93\;\times \;10^{-2}$	−1.2546±0.83673
GFCC_4	$8.29\;\times \;10^{-1}\pm 3.818\;\times \;10^{-1}$	$7.82\;\times \;10^{-1}\pm 8.73\;\times \;10^{-2}$	0.85812±0.43814
GFCC_5	$7.0217\;\times \;10^{-1}\pm 3.312\;\times \;10^{-1}$	$1.06\;\times \;10^{-1}\pm 6.22\;\times \;10^{-2}$	−0.786189±0.3036
GFCC_6	$2.518\;\times \;10^{-1}\pm 2.127\;\times \;10^{-1}$	$6.75\;\times \;10^{-2}\pm 4.55\;\times \;10^{-2}$	0.5097±0.1516
GFCC_7	$-1.938\;\times \;10^{-1}\pm 1.329\;\times \;10^{-1}$	$-1.69\;\times \;10^{1}\pm 1.43$	−0.23444±0.1582
GFCC_8	$3.90\;\times \;10^{-2}\pm 1.230\;\times \;10^{-1}$	$5.24\pm 8.27\;\times \;10^{-1}$	0.06708±0.1302
GFCC_9	$-1.071\;\times \;10^{-1}\pm 8.980\;\times \;10^{-2}$	$-9.81\;\times \;10^{-1}\pm 1.01$	−0.1885±0.0957
GFCC_10	$1.294\;\times \;10^{-2}\pm 7.857\;\times \;10^{-2}$	$9.97\;\times \;10^{-1}\pm 3.55\;\times \;10^{-1}$	$7.4638\;\times \;10^{-2}\pm 7.218\;\times \;10^{-2}$
GFCC_11	$-6.509\;\times \;10^{-2}\pm 5.778\;\times \;10^{-2}$	$-7.78\;\times \;10^{-1}\pm 3.77\;\times \;10^{-1}$	$-3.019\;\times \;10^{-2}\pm 6.7388\;\times \;10^{-2}$
GFCC_12	$5.228\;\times \;10^{-3}\pm 6.801335\;\times \;10^{-2}$	$2.82\;\times \;10^{-1}\pm 1.59\;\times \;10^{-1}$	$3.5798\pm 4.61594\;\times \;10^{-2}$
GFCC_13	$-6.276\;\times \;10^{-2}\pm 4.806\;\times \;10^{-2}$	$-2.05\;\times \;10^{-1}\pm 8.46\;\times \;10^{-2}$	$-5.3772\;\times \;10^{-2}\pm 3.463\;\times \;10^{-2}$
